# Effective Pore Distribution and Mechanism of CO_2_/CH_4_ Dynamic Separation by Carbon Molecular Sieves

**DOI:** 10.3390/nano15211685

**Published:** 2025-11-06

**Authors:** Jianhong Gu, Ran Xu, Zhenlong Song, Zejun Xiao, Shengli Guo, Weile Geng, Xuefu Xian

**Affiliations:** 1 School of Safety and Management Engineering, Hunan Institute of Technology, Hengyang 421002, China; 19183042581@163.com (J.G.); zjxiao@hnit.edu.cn (Z.X.); sliguo_qz@163.com (S.G.); gwle0418@163.com (W.G.); 2 High Performance Computing Department, National Supercomputing Center in Shenzhen, Shenzhen 518055, China; zhenlongsong@alu.cqu.edu.cn; 3 State Key Laboratory of Coal Mine Disaster Dynamics and Control, School of Resources and Safety Engineering, Chongqing University, Chongqing 400044, China; xianxf@cae.cn

**Keywords:** carbon molecular sieve, biogas upgrading, cooperative separation, pore-size distribution, mesopore window

## Abstract

Addressing the pressing demand for biogas and landfill-gas upgrading within the global energy transition, this work strategically combines thermodynamic and kinetic separation principles to identify, from a cooperative-separation perspective, the effective pore-size range that governs carbon molecular sieve (CMS) performance. Thirty anthracite-derived CMS samples with distinct pore structures were synthesized and employed as a statistical set to link pore architecture with dynamic adsorption performance. The results clarify the effective pore-size range and mechanism for enhanced CMS selectivity: CH_4_ uptake depends exclusively on ultramicropores (<10 Å), with a negligible contribution from mesopores (>20 Å), whereas CO_2_ uptake is less sensitive to pore-size distribution. CO_2_/CH_4_ separation performance improves linearly with the volume fraction of mesopores >20 Å, defining a 20–60 Å mesopore window as optimal for cooperative CMS. Mechanistic studies show that a high mesopore fraction significantly slows CH_4_ adsorption while maintaining a fast CO_2_ uptake, thereby amplifying their intrinsic adsorption-rate difference. This work breaks from the conventional purely thermodynamic or kinetic sieving paradigm and offers new design criteria for CMS tailored to on-site biogas and landfill-gas purification.

## 1. Introduction

Carbon molecular sieves are extensively utilized for CO_2_/CH_4_ separation in landfill gas and biogas purification due to their high selectivity, low regeneration energy requirements, robust stability, and low cost [1,2,3,4,5,6,7]. Their separation efficiency stems from the synergy between adsorption mechanisms and material properties, leveraging the differential adsorption of CO_2_ and CH_4_ within pore network, often under low-pressure conditions [8]. During the dynamic separation of CO_2_/CH_4_ mixtures, the pore structure characteristics of CMS play a decisive role [9]. Understanding the effective pore architecture and separation mechanisms of CMS is essential for optimizing performance and guiding the design of application-specific materials for industrial deployment.

Several studies have investigated the effective pore size range for CO_2_/CH_4_ separation, with most focusing on adsorbents developed based on thermodynamic effects [10]. This preference arises from the relatively high quadrupole moment and polarizability of CO_2_, which results in stronger interactions with adsorbent surfaces compared to CH_4_ [11]. Enhancing the surface polarity of adsorbents through chemical modification has proven effective in improving selectivity. For example, Li et al. [12] synthesized nitrogen-doped porous carbon spheres (ACSs-N) from biomass-derived glucose and urea. At 25 °C and 1 bar, ACSs-N exhibited CO_2_ and CH_4_ uptake capacities of 3.03 and 1.30 mmol g^−1^, respectively, with an IAST-predicted CO_2_/CH_4_ (10/90) selectivity of 7.49. Similarly, Deneb et al. [13] modified commercial activated carbon CNR-115 using ammonia, and the resulting CMSCNR-115am demonstrated a significantly enhanced selectivity of up to 129. Thermodynamic CMS materials achieve selective separation by maximizing the difference in CO_2_ and CH_4_ adsorption capacities [14], typically utilizing microporous structures that enhance gas storage potential. Introducing polar surface functionalities within micropores has been shown to further improve selectivity [1]. However, although the effectiveness of thermodynamic strategies in improving selectivity, increased surface polarity can also lead to a moderate increase in CH_4_ uptake. This may compromise the one-pass purification efficiency in industrial settings, often requiring multi-stage enrichment to obtain high-purity gas streams [15,16]. Such limitations remain a challenge for thermodynamic-based adsorbents.

To enhance CO_2_ adsorption performance while effectively suppressing CH_4_ uptake, CMSs based on kinetic separation principles have been developed [17]. The core mechanism of a kinetic CMS relies on exploiting the differences in the diffusion rates of gas molecules within the pore channels [18]. Studies have shown that micropores in the range of 0.33–0.5 nm facilitate the rapid diffusion of CO_2_ while restricting CH_4_ penetration, thereby enabling separation dominated by kinetic selectivity [9]. In contrast, pores with diameters of 0.5–0.8 nm enhance CH_4_ adsorption but reduce selectivity [19,20]. A high proportion of micropores within the 0.33–0.5 nm range is considered optimal for effective CO_2_/CH_4_ separation [21]. The diffusion time constant of CO_2_ is two orders of magnitude greater than that of CH_4_ through the use of highly uniform and tortuous pore pathways to avoid pore mouth blocking or diameter expansion, which can compromise separation performance [22]. A CMS developed based on this steric hindrance–diffusion synergistic mechanism imposes strict structural requirements on the pore network and typically exhibits lower overall adsorption capacities. Consequently, the fabrication of a purely kinetic CMS remains technically challenging.

CMSs relying solely on thermodynamic or kinetic separation principles face inherent limitations, hindering the concurrent optimization of preparation feasibility and separation efficiency. Since both molecular size and polarizability follow the same order for CO_2_ and CH_4_, their thermodynamic and kinetic separation sequences are aligned [11,21]. Therefore, under the premise of thermodynamic adsorption, identifying a pore-size range that significantly amplifies the CO_2_/CH_4_ adsorption-rate difference offers a viable strategy for balancing synthesis complexity with separation performance. Such a thermodynamic–kinetic cooperative CMS relaxes the need for stringent pore-size control, simplifies preparation, and achieves both high separation efficiency and a high adsorption capacity. However, the effective pore-size window for cooperative CMS in CO_2_/CH_4_ separation has not yet been systematically established or discussed.

**Figure 1:**
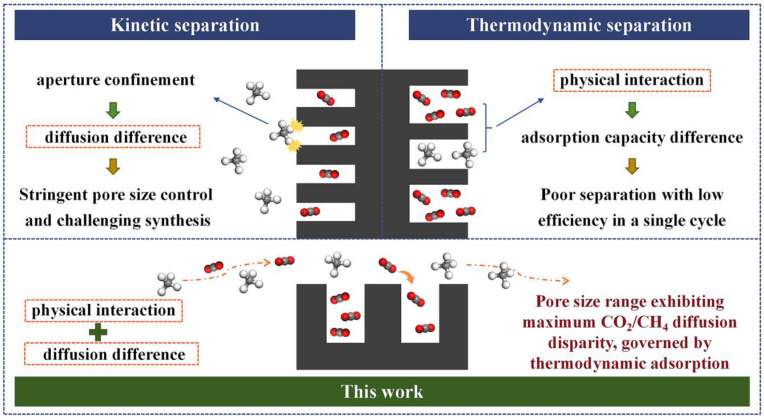
Schematic overview of the prevailing mechanisms.

In this work, the present study develops 30 anthracite-derived CMS samples exhibiting varying CO_2_/CH_4_ selectivities and distinct pore structure distributions, based on the principle of synergistic thermodynamic and kinetic regulation. A statistical analysis approach is employed to evaluate the influence of pore structure on separation performance, with the aim of identifying the effective pore size range for CO_2_/CH_4_ separation. This work breaks away from the conventional paradigm of designing a CMS based solely on thermodynamic or kinetic principles, proposing instead a cooperative strategy that leverages thermodynamic adsorption while amplifying the CO_2_/CH_4_ adsorption-rate difference (Figure 1). It further identifies the pore-size range governing this kinetic disparity, providing theoretical support for the industrial application of a CO_2_/CH_4_-selective CMS and laying a foundation for enhancing biogas and landfill gas utilization, advancing green energy, and accelerating the global energy transition.

## 2. Experimental and Methods

### 2.1. Sample Preparation

A series of CMS samples were synthesized from anthracite coal using starch as a binder, employing CO_2_-based physical activation under systematically varied conditions. Anthracite coal was sourced from Yunnan, China. Soluble starch (analytical reagent grade) was purchased from Tianjin Dingshengxin Chemical Co., Ltd. (Tianjin, China). Air (21% O_2_ + 79% N_2_) and CO_2_ (99%) were supplied by Chongqing Jiaqing Gas Co., Ltd. (Chongqing, China).

Initially, anthracite was crushed and sieved to a particle size below 96 μm. A starch solution was prepared by adding an appropriate amount of water to the starch and stirring until a milk-like suspension was formed. The mixture was then heated on an electric furnace with continuous stirring until the starch underwent gelatinization. The gelatinized starch slurry was subsequently blended with the milled coal and additional water to form a viscous mixture. The resulting paste was extruded into cylindrical pellets (diameter: 1.5 mm; length: 5 mm) using a laboratory-scale forming device. A total of 50 g of the formed pellets was placed in a muffle furnace and subjected to pre-oxidation in air at 573 K for 1 h. Immediately following pre-oxidation, the air supply was cut off, and the temperature was raised to 873 K for carbonization and maintained for 2.5 h. The temperature was then further increased to the activation range, during which CO_2_ was introduced at a controlled flow rate to activate the samples. After activation, the samples were allowed to cool naturally to room temperature. The detailed activation conditions for each sample are provided in Table S1 of the Supplementary Materials. The resulting CMS materials were designated as D1-D30.

To ensure representative benchmarking of adsorption performance, three commercial activated carbon samples were procured from reputable suppliers: Ningxia Huahui Environmental Protection Technology Co., Ltd. (Yinchuan, China), Zhengzhou Clean Water Purification Materials Co., Ltd. (Zhengzhou, China), and Changxing Shanli Chemical Materials Technology Co., Ltd. (Huzhou, China). These commercial samples were designated as the control group and labelled NX-1, ZZ-1, and CX-1, respectively.

### 2.2. Testing and Analysis Procedures

**Figure 2:**
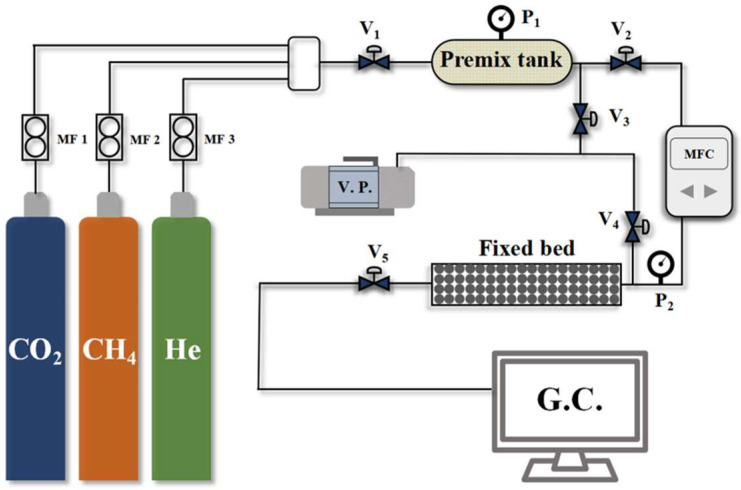
Penetration curve testing apparatus flowchart.

A self-assembled fixed-bed breakthrough apparatus was utilized to evaluate the CO_2_/CH_4_ selectivity of the CMS samples. Compared to simple thermodynamic or kinetic adsorption tests, this method provides a more realistic assessment of the dynamic separation performance of CMS materials. A schematic diagram of the apparatus is shown in Figure 2, with the fixed-bed dimensions and packing details summarized in Table 1. The breakthrough test procedure is as follows:

- Samples were initially dried at 353 K for 6 h in a drying oven to prepare for adsorption measurements. Subsequently, 20 g of the dried sample was loaded into the fixed-bed adsorption column;
- Prior to testing, the system was evacuated under vacuum for 1 h to remove residual impurities. Following evacuation, all valves were closed;
- Feed gas valve V_1_ was opened to introduce pure component gases into the mixing tank, generating a pre-mixed gas with a CO_2_/CH_4_ ratio of 19:81;
- Valve V_2_ was then opened to feed the mixed gas into the fixed bed at a flow rate of 40 mL min^−1^ for the breakthrough test. Valves V_5_ was opened to direct the outlet gas from the adsorption column to a gas chromatograph for continuous analysis of the component concentrations.

**Figure 3:**
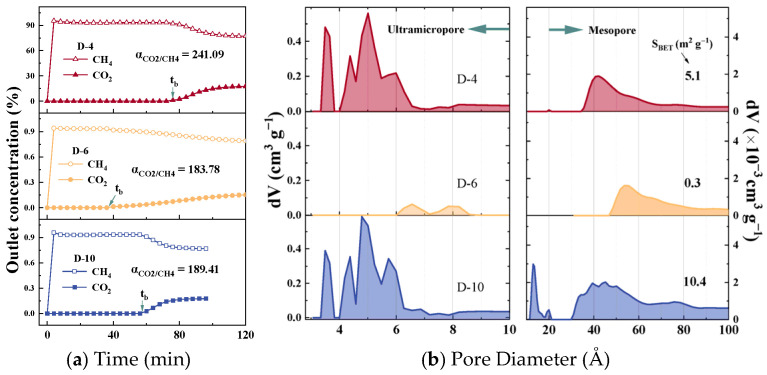
(**a**) Mixed-gas breakthrough curves and (**b**) pore size distribution of D-10, D-6, and D-20.

CH_4_ was the first component to elute from the column, followed by the breakthrough of CO_2_ after a certain duration, marking the end of the experiment. The breakthrough time (*t_b_*) is defined as the time at which the CO_2_ concentration at the column outlet reaches 1% of its inlet concentration (Figure 3a). The dynamic adsorption capacity (*n_i_*) of each component within the breakthrough time was calculated using the following equation [19]:

(1) $$n_{i}=\frac{FC_{0i}}{m}\int_{0}^{t_{b}}\left(1-\frac{C_{i}}{C_{0i}}\right)dt$$

In the equation, *F* represents the initial gas flow rate in L min^−1^; *C*_0_*_i_* and *C_i_* denote the inlet and outlet concentrations of component *i*, respectively, in mol L^−1^; *m* is the mass of the adsorbent in kg; and *t_b_* is the breakthrough time. By substituting the mole fraction of component *i* (*y_i_*) into the expression, the equation can be reformulated as follows:

(2) $$n_{i}=\frac{FC_{0}}{m}\left(y_{i}t_{s}-\int_{0}^{t_{b}}y_{i}dt\right)$$

*C*_0_ represents the inlet gas concentration, expressed in mol L^−1^.

The dynamic separation factor *α_i/j_* can be derived from the dynamic adsorption capacities and is calculated as follows [19]:

(3) $$\alpha_{i/j}=x_{i}y_{j}/y_{i}x_{j}$$

In the equation, *i* and *j* refer to the gas components, *x* denotes the adsorbed-phase concentration, and *y* represents the corresponding gas-phase concentration.

The dynamic adsorption capacity *n_i_* is adopted as the criterion for evaluating the adsorption performance of CMS, while the dynamic separation factor *α* is employed as the key indicator of its separation efficiency.

### 2.3. Pore Structure Characterization

The pore-structure parameters of all samples were characterized using a NOVA Touch LX2 automated surface area and pore-size analyzer (Quantachrome Instruments, Boynton Beach, FL, USA). N_2_ and CO_2_ adsorption–desorption isotherms were measured at 77 K and 273 K, respectively. Before analysis, each sample was degassed under vacuum at 573 K for 3 h to remove surface impurities. The BET-specific surface area (S_BET_) and micropore volume were determined using the multipoint Brunauer–Emmett–Teller method and the t-plot method, respectively. Pore-size distributions were obtained based on quenched-solid density functional theory (QSDFT), which accounts for surface roughness and energetic heterogeneity in porous materials, providing a more accurate depiction of adsorption and capillary phenomena in micro- and mesopores than conventional models. Given the focus on CMS pore characteristics, QSDFT was selected over standard non-local density functional theory (NLDFT) for analyzing samples with complex pore networks [23,24]. The total pore volume (V_t_) was recorded at a relative pressure of 0.98. Due to the critical role of V_t_ in the subsequent statistical analysis, it was also calculated by integrating the cumulative pore volume from the QSDFT PSD for verification. As shown in Figure S1, the two methods yielded nearly identical V_t_ values, confirming the reliability of either approach in statistical evaluation. The average pore diameter (APD) was derived from QSDFT-based pore volume and surface area data.

### 2.4. Pearson Correlation Analysis

Pearson correlation analysis is a statistical method used to evaluate the strength and direction of the linear relationship between two continuous variables [25,26,27]. It is based on the Pearson correlation coefficient, commonly denoted as “P”. This method is widely employed across various disciplines, including scientific research, social sciences, medicine, and economics.

The Pearson correlation coefficient ranges from −1 to +1. A value of +1 indicates a perfect positive linear correlation, meaning that as one variable increases, the other increases proportionally. A value of −1 signifies a perfect negative linear correlation, where an increase in one variable corresponds to a proportional decrease in the other. A value of 0 implies no linear relationship between the variables.

The steps involved in Pearson correlation analysis are as follows: a. collect data for the two variables and calculate their respective means; b. determine the product of the deviations of each data point from the mean, then sum these products; c. calculate the sum of squares of the deviations for each variable; d. divide the total deviation product obtained in step b by the square root of the product of the sums from step c. The resulting value is the Pearson correlation coefficient, P.

By applying Pearson correlation analysis, it is possible to determine whether there exists a statistically significant relationship between the separation performance of CMS and its pore structure characteristics, as well as the strength and direction of such a relationship.

## 3. Results and Discussion

### 3.1. Sample Analysis

Thirty CMS samples were prepared and used as the statistical dataset to investigate the effective pore size range for CO_2_/CH_4_ dynamic separation in thermodynamic–kinetic synergistic systems. To verify the representativeness and stratification of the statistical samples, their separation performances were compared with those of three commercial CMS products: NX-1, ZZ-1, and CX-1. The CO_2_ and CH_4_ adsorption isotherms for the commercial CMSs and three representative synthesized samples (D-4, D-6, D-10) were obtained (Figure S2) and analyzed via Langmuir model fitting (Table S2). The equilibrium selectivity (S_CO2/CH4_) was calculated using the Ideal Adsorbed Solution Theory (IAST), with the detailed procedure provided in the Supplementary Materials. As listed in Table 2, D-6 demonstrated the optimal equilibrium separation performance, with an S_CO2/CH4_ of 10.98, followed by D-4 and D-10, with values of 3.21 and 2.93, respectively, all of which are higher than that of the commercial CMS. Over half of the statistical set demonstrated separation efficiency comparable to that of commercial carbon molecular sieves, while the remainder showed moderate performance, collectively reflecting a representative distribution of separation capabilities across a hierarchical range. Overall, the sample selection in this section is deemed appropriate, and the statistical findings derived from this dataset are considered to be highly reliable.

The CO_2_/CH_4_ mixed-gas breakthrough curves for samples D-4, D-6, and D-10 are presented in Figure 3a. Upon initial exposure, CH_4_ eluted rapidly from the fixed-bed system, whereas CO_2_ reached the outlet only after a discernible adsorption period. This behaviour confirms that the three representative CMS samples exhibit negligible CH_4_ uptake under dynamic flow conditions while selectively retaining CO_2_, thereby enabling the single-pass production of high-purity CH_4_. Calculation of the dynamic separation factor (α) from these curves revealed that D-4 exhibited the highest value at 241.09, followed by D-10 (189.41) and D-6 (183.78). The α of the remaining samples in this work is listed in Table S3. This observed disparity between the actual dynamic separation performance and the IAST-predicted selectivity underscores the necessity of identifying the effective pore-size range that governs the dynamic separation behaviour of CMS materials. Figure 3b shows the pore-size distribution curves of CMS samples D-6, D-10, and D-20, studied for CO_2_/CH_4_ separation, with the remaining samples shown in Figure S3 of the Supplementary Materials. The portion above 10 Å was derived from N_2_ adsorption–desorption isotherms, while the sub-10 Å region was obtained from CO_2_ adsorption–desorption isotherms. The CMS samples developed in this work possess a broader pore-size distribution than most analogues reported in the literature, covering the full spectrum of pore sizes relevant to both purely kinetic and thermodynamic separation mechanisms, thereby enabling a comprehensive statistical assessment of pore-performance relationships. Based on the N_2_-derived pore size distribution, the full pore range was categorized into three segments—>20 Å, 10–20 Å, and <10 Å–for subsequent correlation analysis. The specific surface areas of the CMS samples range from 0.1 to 428.1 m^2^ g^−1^. The minimal values result from excessively mild activation conditions, which caused limited carbon framework disruption, promoted graphitization, and significantly reduced porosity. Detailed values of S_BET_ and V_t_ are provided in Table S3. The <10 Å region obtained from the CO_2_ isotherms was further subdivided based on the distinct peaks observed in Figure 3. Three primary features were identified: a sharp unimodal peak centred around 3.5 Å, a strong continuous peak in the 4–7 Å range, and a weaker continuous distribution between 7 and 10 Å. Accordingly, the sub-10 Å region was divided into three narrower intervals—<4 Å, 4–7 Å, and 7–10 Å—for refined correlation analysis aimed at identifying the optimal pore size range. The CMS samples exhibited considerable variability in their pore structure characteristics, indicating a high degree of diversity within the statistical dataset.

### 3.2. Correlation of CH_4_ Adsorption Capacity

**Figure 4:**
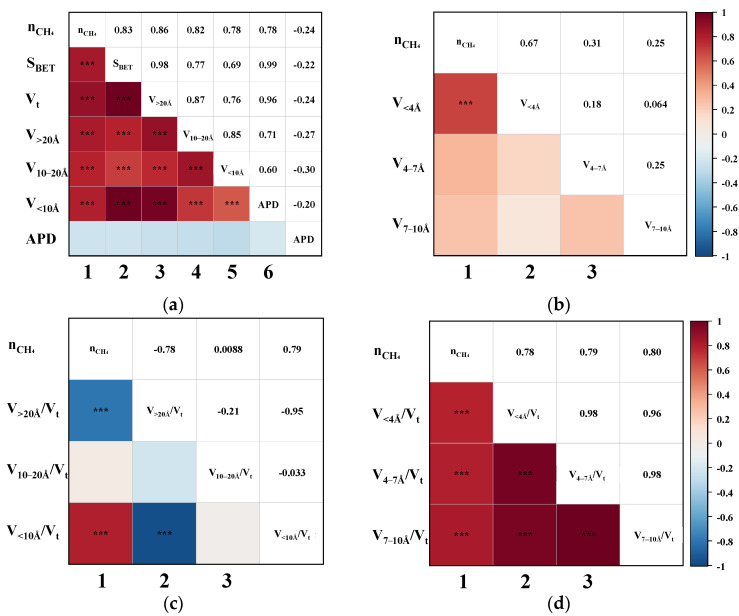
The Pearson correlation triangle plot between nCH_4_ and (**a**) the S_BET_, V_t_, V_>20Å_, V_10–20Å_, V_<10Å_, and APD; (**b**) the V_<4Å_, V_4–7Å_, and V_7–10Å_; (**c**) the proportion of V_>20Å_, V_10–20Å_, and V_<10Å_; (**d**) the proportion of V_<4Å_, V_4–7 Å_, and V_7–10Å_. (“***” indicates that the correlation is highly significant.)

Pearson correlation analysis was performed to evaluate the bivariate relationships between CH_4_ uptake and various pore structure parameters, including S_BET_, V_t_, APD, and segmented pore volumes. Figure 4 illustrates the triangular correlation heatmap between CH_4_ adsorption capacity (nCH_4_) and the structural parameters derived from the CO_2_/CH_4_ breakthrough experiments. In the heatmap, red and blue tiles represent positive and negative correlations, respectively, with colour intensity indicating correlation strength. Asterisks (*) denote statistical significance levels, where more asterisks correspond to higher significance. Figure 4a,b show that the first column corresponds to bivariate correlations between nCH_4_ and each pore structure parameter. The remaining tiles depict intercorrelations among the structural parameters themselves. The heatmap is symmetrical about the diagonal axis, where mirrored positions reflect identical Pearson correlation coefficients (P). For instance, the P for the correlation between nCH_4_ and S_BET_ is 0.83, while that between nCH_4_ and V_t_ is 0.86.

As shown in the first column of Figure 4a, the P between nCH_4_ and S_BET_, V_t_, V_>20Å_, V_10–20Å_, and V_<10Å_ all exceed 0.78, indicating strong positive correlations. Upon further examination of the sub-10 Å ultramicropores in Figure 4b, it is evident that the pores < 4 Å exhibit an even more significant correlation with nCH_4_, suggesting that ultramicropores within this range play a critical role in enhancing CH_4_ adsorption. Overall, pores across the entire measured range appear to be favourable for CH_4_ uptake. To further delineate the most effective pore size range for CH_4_ adsorption, the correlations between the ratio of segmented pore volume to Vt and nCH_4_ were analyzed. This approach is based on the premise that the higher the proportion of truly effective pores, the better the expected separation performance of the CMS [19]. As illustrated in the first column of Figure 4c, nCH_4_ exhibits a strong negative correlation with V_>20Å_/V_t_ (*p* = −0.78), no apparent correlation with V_10–20Å_/V_t_, and a strong positive correlation with V_<10Å_/V_t_ (*p* = 0.79). These findings clearly indicate that only pores smaller than 10 Å contribute positively to CH_4_ uptake. Moreover, Figure 4d reveals that the ratios V_<4Å_/V_t_, V_4–7Å_/V_t_, and V_7–10Å_/V_t_ are all significantly positively correlated with nCH_4_, with *p*-values of 0.78, 0.79, and 0.80, respectively. This suggests that a higher proportion of pores within each of these sub-10 Å segments contributes substantially to CH_4_ adsorption, thereby confirming that increasing the content of <10 Å micropores enhances the overall CH_4_ uptake capacity of CMS materials.

The dynamic adsorption performance of CH_4_ was highly dependent on the pore structure characteristics of the CMS. Specifically, pores smaller than 10 Å exhibited the strongest affinity for CH_4_, while those in the 10–20 Å range showed weaker adsorption, and pores larger than 20 Å contributed least. An excessive proportion of >20 Å pores was detrimental to CH_4_ uptake. The optimal pore size range for CH_4_ adsorption was determined to lie between 4 and 10 Å. These results imply that, in practical dynamic separations, reducing the content of sub-10 Å pores, particularly those favourable to CH_4_ adsorption, could suppress CH_4_ uptake and consequently enhance the concentration of the target gas in a single-stage purification.

### 3.3. Correlation of CO_2_ Adsorption Capacity

**Figure 5:**
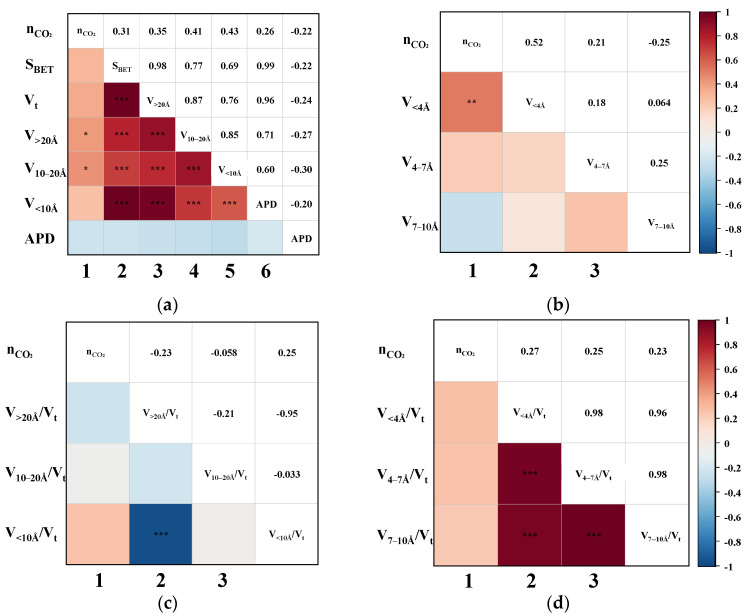
The Pearson correlation triangle plot between nCO_2_ and (**a**) the S_BET_, V_t_, V_>20Å_, V_10–20Å_, V_<10Å_, and APD; (**b**) the V_<4Å_, V_4–7Å_, and V_7–10Å_; (**c**) the proportion of V_>20Å_, V_10–20Å_, and V_<10Å_; (**d**) the proportion of V_<4Å_, V_4–7 Å_, and V_7–10Å_. (The blocks without “*” denote no correlation. “*” indicates slight correlation; “**” indicates moderate correlation; “***” indicates that the correlation is highly significant.)

Having established that the effective adsorption region for CH_4_ lies within pores smaller than 10 Å, further analysis of the pore sizes affecting CO_2_ adsorption helps identify the specific ranges exhibiting the greatest adsorption selectivity contrast. Accordingly, the relationship between CO_2_ uptake and pore structure parameters was assessed, and the resulting triangular correlation diagram is presented in Figure 5. Notably, CO_2_ uptake shows a negligible correlation with most pore-structure parameters in the CMS samples. The strongest observed association was with the V_<4Å_, with a Pearson coefficient of 0.52, indicating only a weak positive correlation. This indicates that CO_2_ adsorption on CMS is largely independent of pore size, showing no strong preference for either micropores or mesopores. Nevertheless, the positive correlations of nCO_2_ with V_<4Å_, V_10–20Å_, and V_>20Å_ suggest that a greater total pore volume generally enhances CO_2_ uptake.

In addition, as shown in columns 2–6 of Figure 5a, S_BET_ and V_t_ exhibit strong positive correlations with pore volumes across all size ranges (V_<10Å_, V_10–20Å_, and V_>20Å_), indicating that increases in pore volume regardless of scale consistently lead to increases in both surface area and total pore volume. This coordinated increase in pore volumes stems from the inherent pore-development mechanism in coal-based CMS. During activation, pore evolution typically progresses from small to larger pores, where extended activation leads to the widening of smaller pores into larger ones. This gradual development produces interconnected pore networks rather than uniform structures, making it challenging to fabricate CMS with a narrow, monomodal pore-size distribution. As such, regulating the proportion of effective pores is a more feasible strategy during material synthesis. Given the minimal pore-size dependence of CO_2_ adsorption and the established <10 Å effective range for CH_4_ uptake, increasing the proportion of larger pores (e.g., V_10–20Å_ and V_>20Å_) should enhance selectivity by suppressing CH_4_ adsorption while maintaining CO_2_ capacity, thereby widening the adsorption performance gap between the two gases.

### 3.4. Correlation of CO_2_/CH_4_ Separation Performance

**Figure 6:**
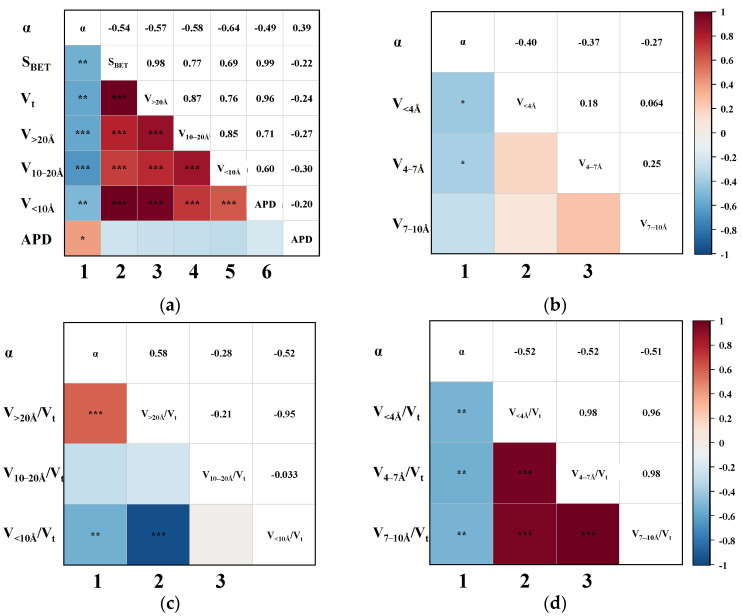
The Pearson correlation triangle plot between αCO_2_/CH_4_ and (**a**) the S_BET_, V_t_, V_>20Å_, V_10–20Å_, V_<10Å_, and APD; (**b**) the V_<4Å_, V_4–7Å_, and V_7-10Å_; (**c**) the proportion of V_>20Å_, V_10–20Å_, and V_<10Å_; (**d**) the proportion of V_<4Å_, V_4–7 Å_, and V_7–10Å_. (The blocks without “*” denote no correlation. “*” indicates slight correlation; “**” indicates moderate correlation; “***” indicates that the correlation is highly significant.)

To further elucidate the influence of CMS pore structure distribution on dynamic CO_2_/CH_4_ separation performance, and to identify the effective pore range, the Pearson correlation between the αCO_2_/CH_4_ and various pore structural parameters was examined. As shown in the first column of Figure 6a,b, the αCO_2_/CH_4_ is negatively correlated with S_BET_, V_t_, and all segmented pore volumes to varying extents, and exhibits a weak positive correlation with APD. Among these, the most pronounced negative correlations were observed between αCO_2_/CH_4_ and the pore volumes of V_>20Å_ (*p* = −0.58) and V_10–20Å_ (*p* = −0.64). Superficially, this implies that the presence of pores, irrespective of size, tends to diminish CO_2_/CH_4_ separation performance, a conclusion that seems counterintuitive when considering practical adsorption behaviour. This apparent discrepancy may arise from the predominance of ineffective pores, which can obscure the role of effective pore regions. To clarify this, the correlation between the proportion of segmented pore volumes and αCO_2_/CH_4_ was further analyzed. As shown in the first columns of Figure 6c,d, a higher proportion of pores > 20 Å correlates positively with αCO_2_/CH_4_, suggesting that this pore domain contributes favourably to gas separation. In contrast, the relative volumes of pores < 20 Å—particularly those <10 Å—are significantly negatively correlated with αCO_2_/CH_4_. These observations are consistent with earlier gas uptake analyses, where CO_2_ adsorption was shown to be relatively insensitive to pore structure, while CH_4_ adsorption was strongly dependent on pore size. Within the CH_4_-favoured region (<10 Å), enhanced CH_4_ uptake results in reduced separation efficiency due to minimal selectivity. Conversely, in the larger pore regime (>20 Å), CH_4_ adsorption is suppressed, while CO_2_ maintains a relatively high uptake, enhancing the selective adsorption of CO_2_ over CH_4_. Considering that the measured pore size distribution of CMSs in this study largely falls below 60 Å, it can be concluded that pores in the range of 20–60 Å and above are most effective in improving CO_2_/CH_4_ separation based on statistical validation. This finding diverges from conventional thermodynamic or kinetic models that define effective pores based solely on equilibrium or diffusion limitations [27]. This deviation stems from the ability of breakthrough testing to capture the combined effects of thermodynamic adsorption, kinetic constraints, and mass transfer behaviour within fixed-bed systems [28]. As a result, the outcomes derived from these dynamic tests provide a more realistic representation of practical separation performance under industrial conditions [29], thereby providing greater reference value for material design and application.

### 3.5. Mechanism of Effective Pores on Performance

**Figure 7:**
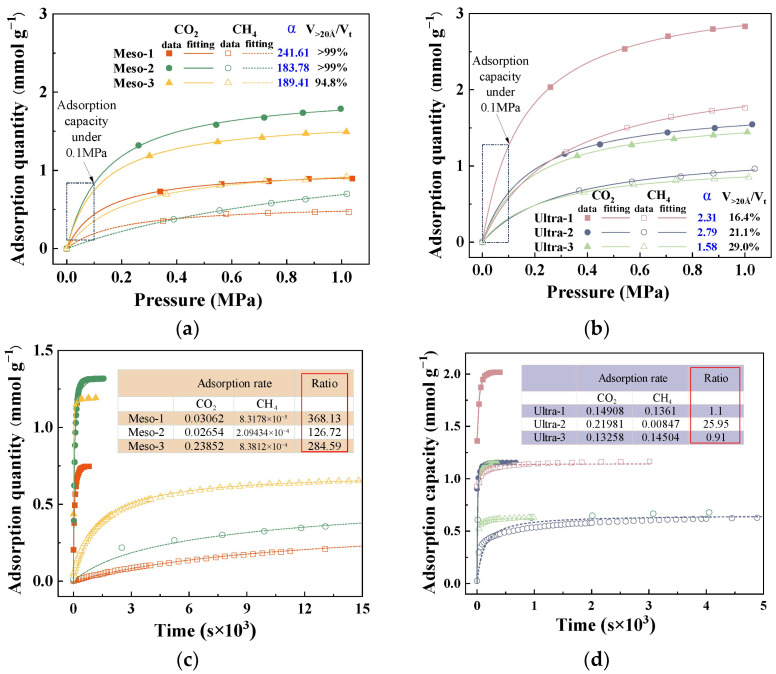
Adsorption isotherms of (**a**) high-ratio mesoporous CMS and (**b**) high-ratio ultramicroporous CMS; kinetic adsorption curves of (**c**) high-ratio mesoporous CMS and (**d**) high-ratio ultramicroporous CMS.

To further clarify how mesopores and larger pores affect the dynamic separation of CO_2_/CH_4_ in CMS, six representative samples were chosen: three with a high proportion of mesopore volume (Meso-1, Meso-2, Meso-3) and three with a high proportion of ultramicropore volume (Ultra-1, Ultra-2, Ultra-3). The adsorption isotherms and kinetic curves of these CMSs were analyzed to examine the influence of effective pore sizes from both thermodynamic and kinetic viewpoints, as shown in Figure 7. All adsorption isotherms exhibited typical Langmuir-type behaviour, suggesting monolayer adsorption on homogeneous surfaces [30,31]. As shown in Figure 7a,b, the Meso-type CMSs showed lower equilibrium adsorption capacities overall for both CO_2_ and CH_4_ compared to the Ultra-type CMSs, attributed to the greater surface area and higher storage capacity offered by micropores at comparable pore volumes. Notably, the difference in gas uptake became more evident at higher pressures. At 0.1 MPa (corresponding to the dynamic adsorption condition), CO_2_ uptake in the Meso group was only slightly lower than in the Ultra group, whereas CH_4_ uptake was substantially reduced. These results suggest that from a thermodynamic standpoint, a higher proportion of mesopores and macropores in CMSs can enhance the selective adsorption of CO_2_ over CH_4_.

**Figure 8:**
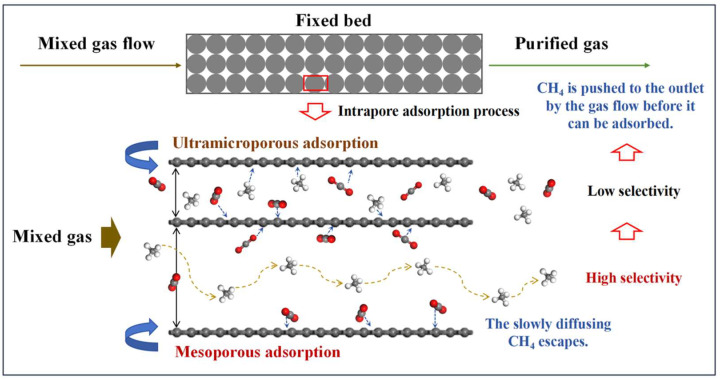
Mechanism governing the influence of effective pore size range.

Figure 7c,d present the kinetic adsorption profiles of the two CMS categories, all well described by the pseudo-first-order kinetic model [32]. The inset table lists the resulting CO_2_ and CH_4_ adsorption rate constants and their ratios. The Meso-series CMS exhibits a significantly greater difference in adsorption rates between CO_2_ and CH_4_ than the Ultra-series, indicating that a high mesopore fraction markedly reduces the CH_4_ adsorption rate (Figure 7c) and hinders CH_4_ from reaching rapid equilibrium. As a result, CH_4_ is typically swept by the flowing stream to the fixed-bed outlet before effective adsorption occurs, as illustrated in Figure 8. When ultramicropores dominate, CMS shows high CH_4_ uptake and similar adsorption rates for both gases, leading to co-adsorption and poor selectivity. In contrast, CMS with prevalent mesopores exhibits low CH_4_ uptake and a slower CH_4_ adsorption rate, allowing CO_2_ to be captured rapidly while CH_4_ is largely purged before adsorption, thereby enhancing selectivity. Therefore, mesopores constitute the effective pore-size window for dynamic CO_2_/CH_4_ separation in CMS, and deliberately increasing the mesopore fraction during synthesis helps produce high-performance materials. However, excessively large pores diminish gas storage capacity, and unlimited pore enlargement would reduce CO_2_ uptake. Based on the pore distributions observed, a pore-size range of 20–60 Å is recommended.

## 4. Conclusions

In this study, CMSs with varied pore structure distributions were prepared from anthracite and systematically characterized. Through integrated fixed-bed breakthrough experiments, QSDFT pore analysis, and Pearson correlation analysis, the effective pore size range and separation mechanism for CO_2_/CH_4_ were clarified from a thermodynamic-kinetic synergy perspective. The results demonstrated that CH_4_ adsorption exhibited a strong positive correlation with ultramicropores < 10 Å, while mesopores > 20 Å contributed negligibly to adsorption and substantially suppressed CH_4_ uptake at high proportions. CO_2_ adsorption showed minimal dependence on pore size while maintaining high capacity across the measured ranges, thereby enabling separation via differential adsorption. By tuning the mesopore proportion in the 20–60 Å range, CH_4_ adsorption can be effectively limited while retaining CO_2_ capacity, achieving high-purity CH_4_ in a single breakthrough. This finding extends beyond conventional micropore-based thermodynamic or kinetic separation strategies and provides a quantifiable design basis for CMSs in practical biogas/landfill gas upgrading, and offering engineering value for clean energy utilization and carbon reduction.

## Figures and Tables

**Table 1:** Characteristics of the fixed bed.

Fixed Bed Specifications	Value
Adsorbent packing mass	20 g
Fixed bed length	0.27 m
Fixed bed inner diameter	0.014 m

**Table 2:** Sample performance evaluation.

Sample	Equilibrium Adsorption Capacity (mmol g^−1^)		S_CO2/CH4_	Notes
	CO_2_	CH_4_		
NX-1	0.80	0.36	2.24	More than half of the samples have an S_CO2/CH4_ above 1.32.
ZZ-1	0.74	0.56	1.32	
CX-1	0.92	0.60	1.75	
D-4	0.78	0.30	3.21	This study
D-6	0.83	0.12	10.98	This study
D-10	0.74	0.33	2.93	This study

## Data Availability

The original contributions presented in this study are included in the article/Supplementary Materials. Further inquiries can be directed to the corresponding author.

## Supplementary Materials

The following supporting information can be downloaded at: https://www.mdpi.com/article/10.3390/nano15211685/s1. Table S1: Sample preparation conditions; Table S2: Langmuir-fitting parameters of the isotherms; Table S3: Pore structure characteristics of the CMS samples; Figure S1: Comparison of total pore volume values derived from the two calculation approaches; Figure S2: Adsorption isotherms of the commercial CMS and the samples in this work. References [2,33,34] are cited in the Supplementary Materials.
