# Supplementary material for: Effective Pore Distribution and Mechanism of CO2/CH4 Dynamic Separation by Carbon Molecular Sieves
Source: Nanomaterials (Basel). 2025 Nov 6;15(21):1685. doi: 10.3390/nano15211685 (PMC12609680; doi:10.3390/nano15211685)
Supplement: Supplementary file 1 [file nanomaterials-15-01685-s001.zip › nanomaterials-3925716-supplementary.pdf]

Table S1. Sample preparation conditions.

Sample	Temperature (K)	Flow rate (L h^{-1})	Time (h)	Sample	Temperature (K)	Flow rate (mL h^{-1})	Time (h)
D-1	973	0	48	D-16	973	36	4
D-2	973	3	24	D-17	1073	24	2
D-3	973	3	48	D-18	1073	24	4
D-4	973	4.8	12	D-19	1073	36	1
D-5	973	4.8	18	D-20	1073	36	2
D-6	973	4.8	24	D-21	1073	36	4
D-7	973	6	24	D-22	1173	24	2
D-8	973	6	48	D-23	1173	24	4
D-9	973	7.2	24	D-24	1173	36	1
D-10	973	9	48	D-25	1173	36	2
D-11	1023	6	48	D-26	1173	36	4
D-12	1073	6	48	D-27	1223	24	2
D-13	973	24	2	D-28	1223	24	4
D-14	973	24	4	D-29	1223	36	2
D-15	973	36	2	D-30	1223	36	4

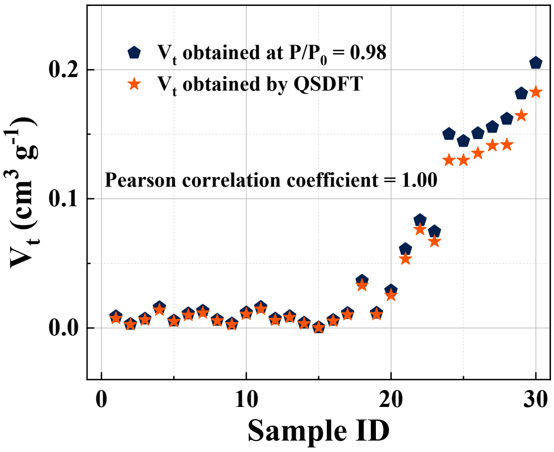

Figure S1. Comparison of total pore volume values derived from the two calculation approaches.

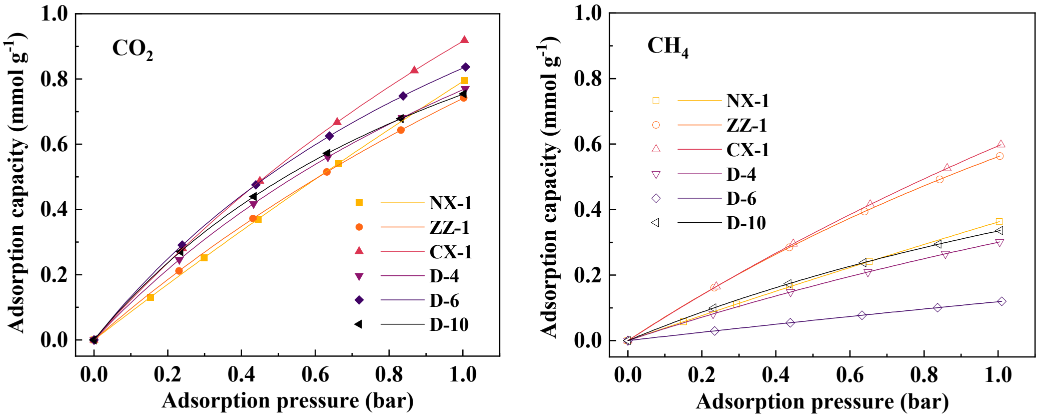

(a) (b)

Figure S2. Adsorption isotherms of the commercial CMS and the samples in this work.

Fitting of adsorption isotherms

The adsorption isotherms of samples are measured at 316.5K. The isotherm data are fitted by Langmuir model as follows [33]:

$$q_i = \frac{q_{mi} b_i p}{1 + b_i p} \tag{S1}$$

where q_i and q_{mi} are the adsorbed amount and the maximum adsorbed amount of gas i, respectively; p is the pressure of the gas; and b_i is the adsorption coefficient. The calculated results of qm and b are listed in Table S1.

The ideal adsorbed solution theory model

The ideal adsorbed solution theory (IAST) model is an important theory for the analysis of multiple systems. Based on the IAST theory, the selectivity of a binary gas mixture can be determined by the equilibrium separation factor (S) between the mixture components within the range of operating pressure and temperature [2]. The calculation formula is as follows:

$$S_{i/j} = \frac{x_i / y_i}{x_j / y_j} \tag{S2}$$

where x_i, y_i and x_j, y_j are the mole fractions of components i and j in the adsorbed and gas phases, respectively.

For adsorption isotherms that follow the Langmuir equation, the S value is a constant [34], and the calculation formula is as follows:

$$S_{i/j} = \frac{q_{mi} b_i}{q_{mj} b_j} \tag{S3}$$

where q_{mi}, q_{mj} and b_i, b_j are the maximum adsorbed amount and adsorption coefficient of gas i and j fitted by Langmuir model.

Table S2. Langmuir-fitting parameters of the isotherms.

Sample	Equilibrium adsorption capacity (mmol g^{-1})		q_m		b		$S_{CO2/CH4}$
	CO_2	CH_4	CO_2	CH_4	CO_2	CH_4	
NX-1	0.80	0.36	8.99449	5.50044	0.96584	0.70453	2.24
ZZ-1	0.74	0.56	2.96937	2.27895	3.3223	3.26871	1.32
CX-1	0.92	0.60	3.26414	3.24239	3.89783	2.24493	1.75
D-4	0.78	0.30	2.12004	1.50617	5.66696	2.48845	3.21
D-6	0.83	0.12	2.02339	1.47578	7.00047	0.8744	10.98
D-10	0.74	0.33	1.66084	1.16311	8.29631	4.03762	2.93

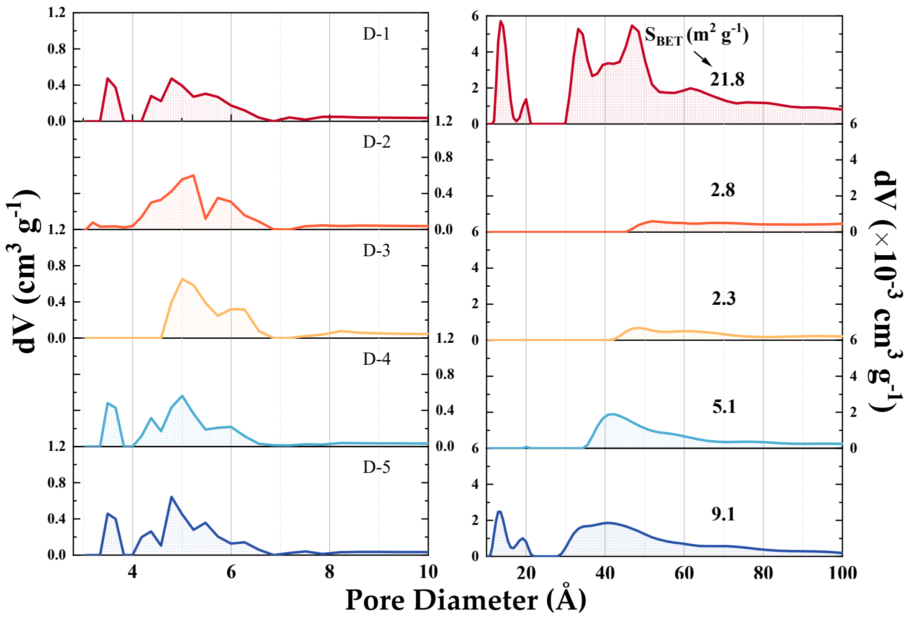

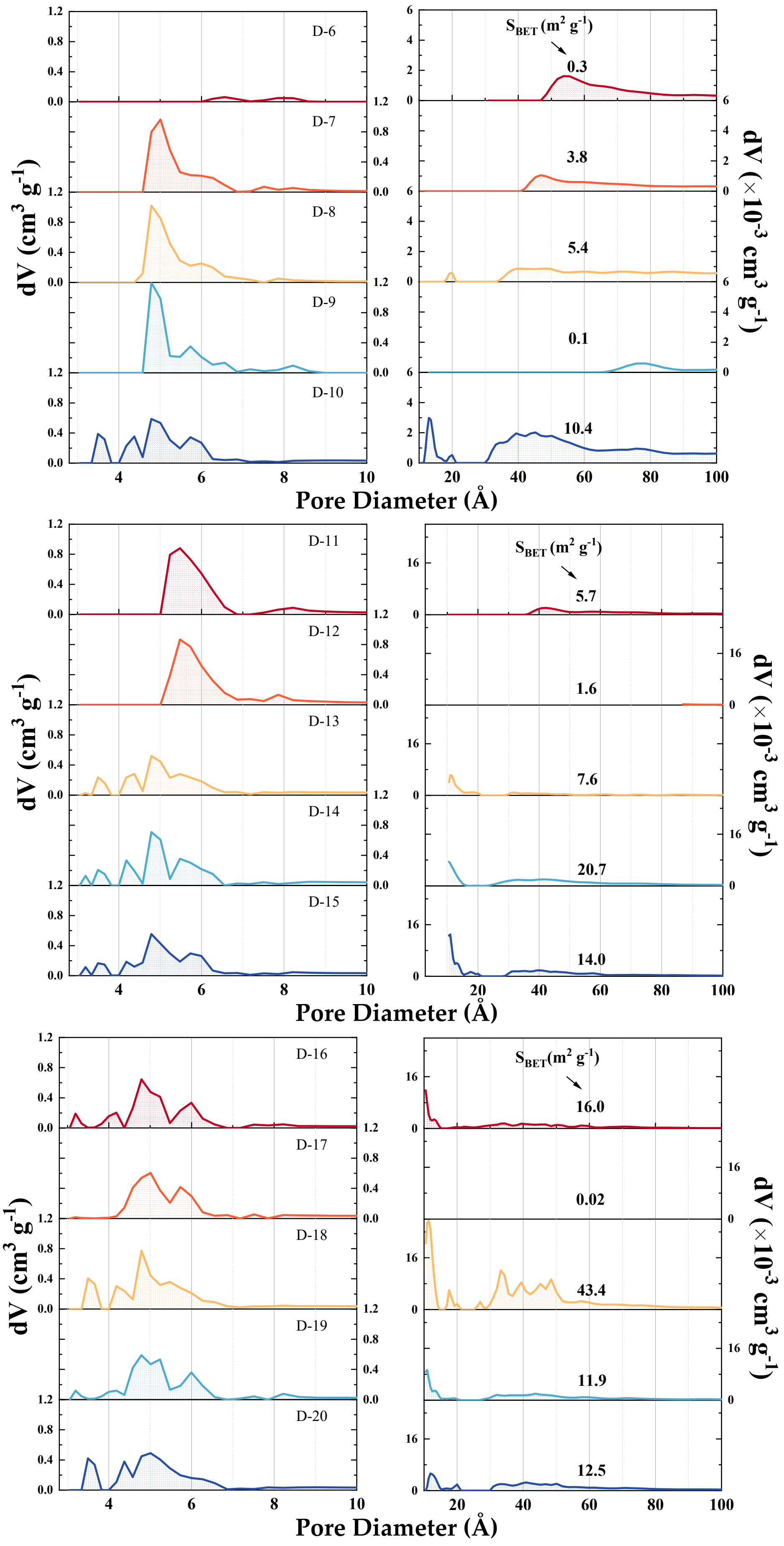

D-6
D-7
D-8
D-9
D-10
S_BET (m2 g-1)
0.3
3.8
5.4
0.1
10.4
dV (cm3 g-1)
dV (×10-3 cm3 g-1)
Pore Diameter (Å)
D-11
D-12
D-13
D-14
D-15
S_BET (m2 g-1)
5.7
1.6
7.6
20.7
14.0
dV (cm3 g-1)
dV (×10-3 cm3 g-1)
Pore Diameter (Å)
D-16
D-17
D-18
D-19
D-20
S_BET(m2 g-1)
16.0
0.02
43.4
11.9
12.5
dV (cm3 g-1)
dV (×10-3 cm3 g-1)
Pore Diameter (Å)

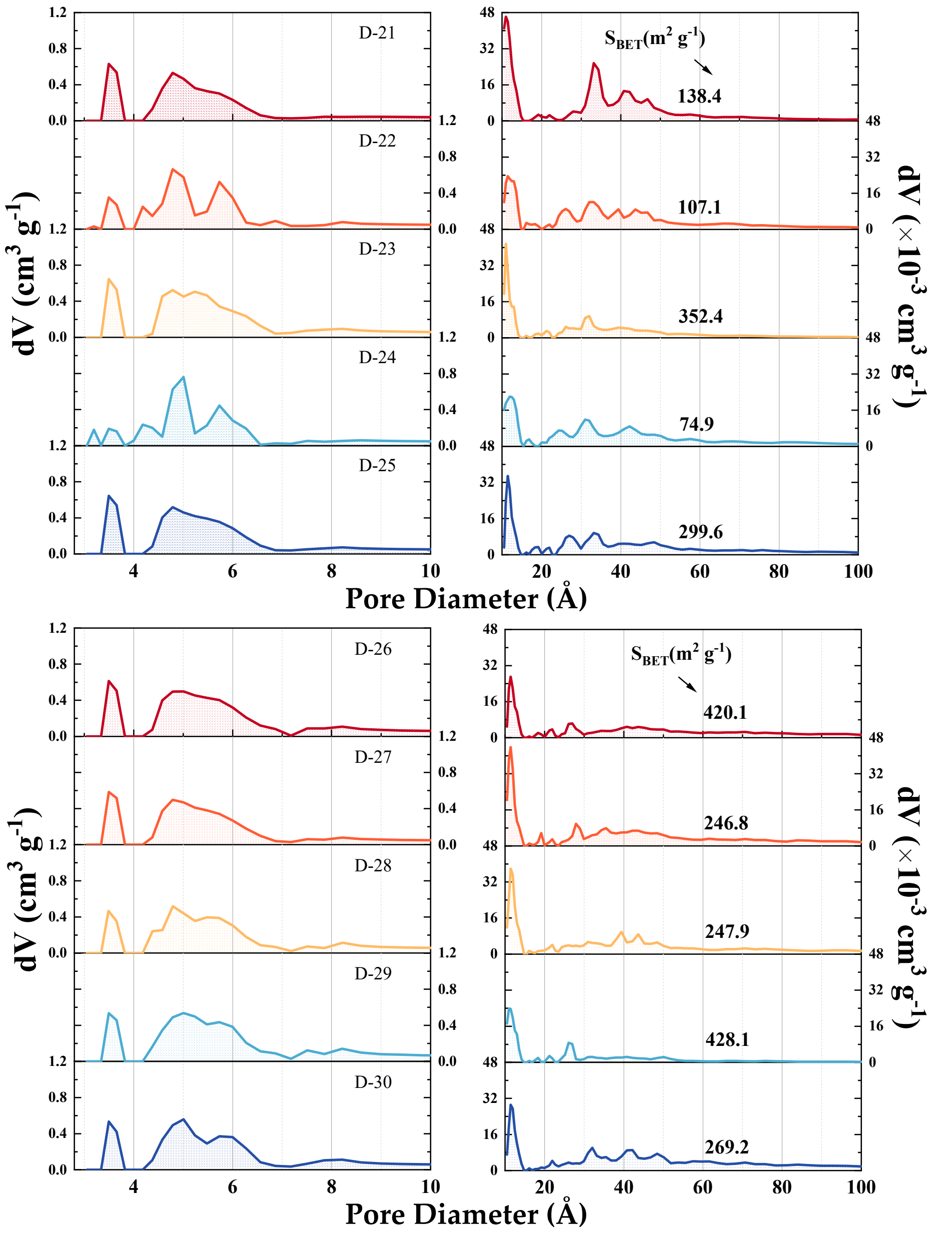

Figure S3. Pore size distribution of the CMS samples.

Table S3. Pore structure characteristics of the CMS samples.

Sample	S_{BET} (m^2 g^{-1})	V_t (cm^3 g^{-1})	$\alpha_{CO2/CH4}$	Sample	S_{BET} (m^2 g^{-1})	V_t (cm^3 g^{-1})	$\alpha_{CO2/CH4}$
D-1	21.81	0.029	7.02	D-16	16.04	0.012	117.37
D-2	2.79	0.007	241.61	D-17	0.2	0.0004	71.25
D-3	2.34	0.004	77.21	D-18	43.38	0.036	10.18
D-4	5.14	0.006	241.09	D-19	11.90	0.012	19.53
D-5	9.08	0.009	83.20	D-20	12.47	0.013	153.04
D-6	0.29	0.006	183.78	D-21	138.39	0.083	6.44
D-7	3.83	0.006	59.60	D-22	107.11	0.075	5.40
D-8	5.37	0.011	164.11	D-23	352.35	0.156	2.79
D-9	0.10	0.003	144.28	D-24	74.92	0.061	6.68
D-10	10.43	0.016	189.41	D-25	299.57	0.151	3.11
D-11	5.70	0.009	4.34	D-26	420.10	0.205	1.58
D-12	1.61	0.003	4.24	D-27	246.82	0.150	4.56
D-13	7.61	0.007	85.29	D-28	247.90	0.145	3.19
D-14	20.70	0.016	103.21	D-29	428.13	0.182	2.31
D-15	14.01	0.012	7.28	D-30	269.24	0.162	2.36

References

Gu, M.; Zhang, B.; Qi, Z.; et al. Effects of pore structure of granular activated carbons on CH_4 enrichment from CH_4/N_2 by vacuum pressure swing adsorption. *Sep. Purif. Technol.* **2015,** 146, 213-218. http://dx.doi.org/10.1016/j.seppur.2015.03.051.

Li, Y.; Xu, R.; Wang, B.; et al. Enhanced N-doped Porous Carbon Derived from KOH-Activated Waste Wool: A Promising Material for Selective Adsorption of CO_2/CH_4 and CH_4/N_2. *Nanomaterials (Basel)* **2019,** 9, 1-15. https://doi.org/10.3390/nano9020266.

Zhang, B.; Huang, Z.; Liu, P.; et al. Influence of pore structure of granular activated carbon prepared from anthracite on the adsorption of CO_2, CH_4 and N_2. *Korean J. Chem. Eng.* **2022,** 39(3), 724-735. https://doi.org/10.1007/s11814-021-0948-4.
